# Supplementary material for: Towards system redesign: An exploratory analysis of neurodivergent traits in a childhood population referred for autism assessment
Source: PLoS One. 2024 Jan 10;19(1):e0296077. doi: 10.1371/journal.pone.0296077 (PMC10781046; doi:10.1371/journal.pone.0296077)
Supplement: Attachment — Submitted filename: Response to Reviewers.docx [file pone.0296077.s002.docx]

# PONE-D-23-18648

# Towards system redesign: an exploratory analysis of neurodivergent traits in a childhood population referred for autism assessment.

Dear Academic Editor and Reviewers,

The authors would like to thank the academic editor and reviewers for their very helpful, insightful and thoughtful suggestions.

In short, we have implemented all suggestions for the resubmitted manuscript attached.

Specifically in response to reviewers comments we have made the following changes:

## Academic Editor -

*Comment 1: “Editor concurs with the observations made by the reviewers regarding Figure 2, which presents challenges in comprehension.”*

This issue was identified by both reviewers and the academic editor. We have addressed this issue by significantly reworking the figure to improve clarity. We redrew the figure using R to improve the proportionality of the diagram. We revised the colouring and added a key to improve visual access. We have relabelled the figure with a more comprehensive explanation of the contents. In addition, we tested our new version of the diagram with multiple members of our team who were not familiar with the content of the paper or previous diagram. They were all were able to understand a derive benefit from the improved version.

*Comment 2: “It is imperative to enhance the clarity of the figure legends, ensuring that they are explicitly labelled in accordance with their respective sequencing”.*

We have addressed this comment by improving the clarity of the figure and table legends throughout the manuscript. Again, we have tested the new labelling with members of our team unfamiliar with the paper and we have further improved the legends following this feedback.

*Comment 3: “In the ensuing discussion, there arises the necessity to incorporate a more substantial body of evidence derived from the extant scholarly literature. Additionally, it is advisable to scrutinize whether any contrasting studies exist in the relevant literature that merit consideration and inclusion within the discourse”.*

The discussion section has been completely overhauled with relevant additional discussion of the literature relevant to our study. We have included further discussion and associated references around what is currently known regarding overlap of the neurotypes which our study addresses. We have also made further searches for contrasting studies. While we were not able to find any studies which disagreed with our findings, our results showed a greater overlap of neurotypes than other similar studies. This has been added to the discussion and we have reflected on some reasons for the potential differences highlighted.

## Reviewer 1 –

*Comment 1: “I found Figure 2 difficult to understand”.*

This figure has been completely reworked in line with the above detailed description.

*Comment 2: “The statement about the selection of median needs a little more explanation. In what ways were the mean scores skewed (line 213-214)”*

We have included further information regarding this. We have added information to the methods and results sections detailing the data skew and justifying the use of the median rather than the mean. In rediscussing with our statistician, their opinion is that using the median is valid, and that by including the nature of the skewed data, this should provide an explanation to the reader as to why the median and not the mean was selected.

*Comment 3: “In the final paragraph Line 418 onwards) the authors may consider recommending a similar study in the adult population as they too are not well served by siloed services, a trend that is increasingly prevalent.”*

This is a very pertinent point which we overlooked in the original paper. We completely agree, and we have added a statement to the conclusion section regarding this.

## Reviewer 2 -

*Comment 1: “Legend of figures need to be more clear and labelled as per sequence.”*

We agree and these have been so revised throughout the paper, including improved descriptors for figure 1 and 2 and in relation to the included tables.

*Comment 2: “2nd figure in the sequence is not clear”.*

This has been fully reworked. See detail response to similar comments received above.

*Comment 3: “Discussion should include more evidence from literature and if there are any contrasting studies?”*

This has been addressed by completely reworking the discussion section to include more relevant and wider reference to the existing literature. In addition, while we did not find any contrasting studies, we did find evidence that our rate of overlap was higher than in some similar studies. We have reflected on some possible reasons for our increased rates and have explicitly discussed this in the discussion section.

*Comment 4: “Page 301: 'only' should be removed”.*

This has been done.

*Comment 5: “Page 338: Replace caseness with 'cases'”.*

This has been done.

*Comment 6: “Use 'DSM-5' instead of 'DSM5' throughout the article”*

This has been changed throughout the article.

Once again, we would like to thank the academic editor and the reviewers for their time in reviewing our article and for the thoughtful and helpful suggestions for improvement. We trust that we have addressed the issues highlighted to your satisfaction however we would be happy to act on any further advice or comment for improvement you may have.

Yours faithfully,

Dr Jason Lang

(On behalf of the authors).
